# Supplementary material for: Evaluating the Histologic Grade of Digital Squamous Cell Carcinomas in Dogs with Dark and Light Haircoat—A Comparative Study of the Invasive Front and Tumor Cell Budding Systems
Source: Vet Sci. 2020 Dec 30;8(1):3. doi: 10.3390/vetsci8010003 (PMC7824281; doi:10.3390/vetsci8010003)
Supplement: Supplementary file 1 [file vetsci-08-00003-s001.pdf]

* u: unknown

	Phenotypical Haircoat color	Genetic group based on genotypic haircoat color	Breed	Age (years)	Sex<br>0= unknown<br>1=female 2=female spayed<br>3=male 4=male castrated
**1**	Black	1a	Briard	11	1
**2**	Black	1a	Briard	7	4
**3**	Black	1a	Giant Poodle	7	4
**4**	Black	1a	Labrador	11	4
**5**	Black	1a	Russian Terrier	6	0
**6**	Black	1a	Russian Terrier	10	2
**7**	Black	1a	Russian Terrier	8	3
**8**	Black	1a	Russian Terrier	8	3
**9**	Black	1a	Russian Terrier	10	1
**10**	Black	1a	Russian Terrier	6	0
**11**	Black	1a	Russian Terrier	10	2
**12**	Black	1b	Giant Schnauzer	9	4
**13**	Black	1b	Giant Schnauzer	11	4
**14**	Black	1b	Giant Schnauzer	11	2
**15**	Black	1b	Giant Schnauzer	10	1
**16**	Black	1b	Giant Schnauzer	9	3
**17**	Black	1b	Giant Schnauzer	10	2
**18**	Black	1b	Giant Schnauzer	10	3
**19**	Black	1b	Giant Schnauzer	8	1
**20**	Black	1b	Giant Schnauzer	8	3
**21**	Black	1b	Giant Schnauzer	9	2
**22**	Black	1b	Giant Schnauzer	9	4
**23**	Black	1b	Giant Schnauzer	10	1
**24**	Black	1b	Giant Schnauzer	6	4

**25**	Black	1b	Giant Schnauzer	10	4
**26**	Black	1b	Giant Schnauzer	8	3
**27**	Black	1b	Giant Schnauzer	7	3
**28**	Black	1b	Giant Schnauzer	9	4
**29**	Black	1b	Giant Schnauzer	10	3
**30**	Black	1b	Giant Schnauzer	8	3
**31**	Black	1b	Giant Schnauzer	11	3
**32**	Black	1b	Giant Schnauzer	6	2
**33**	Black	1b	Giant Schnauzer	10	3
**34**	Black	1b	Giant Schnauzer	11	3
**35**	Black	1b	Giant Schnauzer	12	1
**36**	Black	1b	Giant Schnauzer	8	4
**37**	Black	1b	Giant Schnauzer	13	2
**38**	Black	1b	Giant Schnauzer	7	4
**39**	Black	1b	Standard Schnauzer	10	2
**40**	Black	1b	Standard Schnauzer	8	3
**41**	Black	1b	Standard Schnauzer	8	2
**42**	Black	1b	Standard Schnauzer	13	3
**43**	Black	1b	Standard Schnauzer	13	1
**44**	Black	1b	Standard Schnauzer	12	4
**45**	Black	1b	Standard Schnauzer	10	3
**46**	Black&tan	1c	Gordon Setter	10	2
**47**	Black&tan	1c	Gordon Setter	11	1
**48**	Black&tan	1c	Gordon Setter	12	3
**49**	Black&tan	1c	Gordon Setter	12	3
**50**	Black&tan	1c	Gordon Setter	11	3
**51**	Black&tan	1c	Gordon Setter	11	1

**52**	Black&tan	1c	Gordon Setter	8	3
**53**	Black&tan	1c	Gordon Setter	u	3
**54**	Black&tan	1c	Gordon Setter	u	4
**55**	Black&tan	1c	Gordon Setter	11	3
**56**	Black&tan	1c	Rottweiler	10	1
**57**	Black&tan	1c	Rottweiler	10	0
**58**	Black&tan	1c	Rottweiler	8	2
**59**	Black&tan	1c	Rottweiler	8	3
**60**	Black&tan	1c	Rottweiler	11	0
**61**	Black&tan	1c	Rottweiler	7	3
**62**	Black&tan	1c	Rottweiler	6	1
**63**	Black&tan	1c	Rottweiler	10	1
**64**	Black&tan	1c	Rottweiler	8	2
**65**	Black&tan	1c	Rottweiler	10	2
**66**	Black&tan	1c	Rottweiler	11	2
**67**	Black&tan	1c	Rottweiler	8	2
**68**	Black&tan	1c	Rottweiler	8	4
**69**	Black&tan	1c	Rottweiler	9	3
**70**	Black&tan	1c	Rottweiler	10	2
**71**	Black&tan	1c	Rottweiler	7	3
**72**	Black&tan	1c	Rottweiler	13	1
**73**	Black&tan	1c	Rottweiler	8	2
**74**	Black&tan	1c	Rottweiler	10	1
**75**	Black&tan	1c	Rottweiler	13	4
**76**	Black&tan	1c	Rottweiler	11	3
**77**	Light	2	Golden Retriever	13	3
**78**	Light	2	Golden Retriever	10	1

**79**	Light	2	Golden Retriever	8	3
**80**	Light	2	Golden Retriever	11	3
81	Light	2	Golden Retriever	11	1
82	Light	2	Golden Retriever	9	3
83	Light	2	Golden Retriever	8	1
84	Light	2	Golden Retriever	8	4
85	Light	2	Golden Retriever	12	0
86	Light	2	Golden Retriever	7	3
87	Light	2	Golden Retriever	10	3
88	Light	2	Golden Retriever	14	3
89	Light	2	Golden Retriever	9	0
90	Light	2	Golden Retriever	12	3
91	Light	2	Golden Retriever	12	0
92	Light	2	West Highland White Terrier	12	4
93	Light	2	West Highland White Terrier	12	1
94	Light	2	West Highland White Terrier	12	4
